# Primary eosinophilic gastrointestinal disorders and allergy: Clinical and therapeutic implications

**DOI:** 10.1002/clt2.12146

**Published:** 2022-05-23

**Authors:** Carlo Maria Rossi, Marco Vincenzo Lenti, Stefania Merli, Amelia Licari, Martina Votto, Gian Luigi Marseglia, Antonio Di Sabatino

**Affiliations:** ^1^ First Department of Internal Medicine IRCCS Fondazione Policlinico San Matteo University of Pavia Pavia Italy; ^2^ Department of Clinical‐Surgical, Diagnostic and Pediatric Sciences University of Pavia Fondazione IRCCS Policlinico San Matteo Pavia Italy

**Keywords:** allergy, asthma, atopic dermatitis, food allergy, rhinitis

## Abstract

Primary eosinophilic gastrointestinal disorders (EGID) are increasingly prevalent, immune‐mediated, chronic conditions which primarily affect pediatric and young adult patients, leading to substantial disease burden, and poor quality of life. EGID may either involve single portions of the gastrointestinal tract (i.e., esophagus, stomach, small bowel, and colon) or a combination. Their strong association with allergic disorders has been recently recognized, and although their shared pathophysiological basis remains partly elusive, this feature greatly impacts the diagnostic and treatment work‐up. We herein critically discuss the current knowledge on the association of EGID and allergic disorders, including atopic dermatitis, allergic rhinitis, allergic asthma, and food or drug allergy. In particular, we reviewed the literature focusing on their epidemiology, pathophysiological basis and mechanisms, and diagnostic strategies. Finally, we discuss the currently ongoing clinical trials targeting EGID and allergic diseases, including, among others the monoclonal antibodies dupilumab, mepolizumab, benralizumab, and lirentelimab.

**Abbreviations**
EGID: eosinophilic gastrointestinal disorders
EoE: eosinophilic esophagitis

## 1 INTRODUCTION

Primary eosinophilic disorders of the gastrointestinal tract (EGID) encompass a spectrum of diseases characterized by prominent eosinophilic inflammation affecting different regions of the gut that occur in the absence of secondary causes (e.g., infections, drug reactions).^1^, ^2^ Eosinophils typically show an activated phenotype, and their infiltration leads to symptoms related to organ dysfunction. EGID include some major entities according to the topographical localization of the inflammation, namely eosinophilic esophagitis (EoE), eosinophilic gastritis/gastroenteritis, and eosinophilic colitis, and both the pediatric and adult populations can be affected by these conditions, although with different manifestations in the pediatric and adult populations.^3^, ^4^

Eosinophilic gastrointestinal disorders are increasingly recognized conditions, the prevalence of which has been probably underestimated so far due to poor awareness and lack of standardized diagnostic criteria.^5^, ^6^ Also, given that endoscopic examinations are needed for making a definitive diagnosis, the entity of underdiagnosis in pediatric patients is probably more relevant. More in depth, EoE, with a prevalence of 0.5 to 1/1000 individuals in the general population, is the most frequent among EGID, and hence it is the most studied.^7^ It represents the most common cause of chronic dysphagia in children and the most common cause of dysphagia with bolus impaction in adults.^8^ In a recent study by Cianferoni et al. conducted in the United States, the prevalence of concomitant atopic diseases was significantly higher in both adults and children, compared to non EoE patients.^9^

Due to their supposed rarity and the paucity of data, the prevalence of the other disorders belonging to the EGID spectrum is more difficult to ascertain. According to a recent US registry‐based study by Dellon et al., the prevalence of eosinophilic gastritis, gastroenteritis and eosinophilic colitis, after the introduction of specific ICD‐9 codes, can be estimated to be as high as 6.3/100,000, 8.4/100,000, and 3.3/100,000, respectively.^10^ However, this figure is probably underestimated, as this commonly occurs in administrative data‐driven studies.^11^ As recently reported in a systematic review with meta‐analysis, non‐esophageal EGIDs affect about 2% of patients referred to the hospitals for gastrointestinal symptoms and the prevalence of atopic comorbidities ranges from 25% to 54% of affected patients.^12^

Eosinophilic esophagitis is a chronic immune‐mediated, antigen‐driven, disease, and results from the complex interplay between genetic and environmental factors, also including early life exposures to certain factors, leading to epithelial barrier dysfunction, allergic sensitization, and prominent Th2 inflammation.^8^, ^13^, ^14^ On the contrary, the pathogenesis of EGID not affecting the esophagus is still largely uncertain. Some cellular and molecular features of Th2 inflammation have been demonstrated, particularly with reference to eosinophilic gastroenteritis, but autoimmune factors are also believed to exert a role.^15^ ^,^ ^16^ However, a comprehensive view of their pathogenesis is still lacking, and this contributes, along with other factors, to the substantial diagnostic delay and therapeutic uncertainty.^17^ ^,^ ^18^ Moreover, the association with allergic disorders must be considered when managing patients with EGID, as they may share a common etio‐pathological background and hence some clinical features.^2^, ^9^, ^17^ In fact, some patterns of disease association are common in these patients, such as the co‐occurrence of allergic asthma, rhinitis, and esophageal symptoms, or the occurrence of gastrointestinal symptoms in patients receiving oral immunotherapy for food allergy, or else the occurrence of isolated diarrhea in atopic patients.^6^, ^19^, ^20^ All these clinical patterns should raise the suspicious of EGID.

Apart from the common association with allergic manifestations, the clinical features of EGID vary according to the gut segment and the layer of the gut wall involved, that is, the mucosa, the muscular layer, or the serosa, and the diagnostic work‐up of EGID is primarily based on endoscopy and histopathology.^21^ The main clinical features, diagnostic criteria, and currently available therapies for EGID are summarized in Table 1.

**TABLE 1:** Clinical and endoscopic features, diagnostic criteria, current therapeutic options in eosinophilic gastrointestinal disorders

	Eosinophilic esophagitis	Eosinophilic gastritis/enteritis	Eosinophilic colitis
Clinical features	Symptoms vary with age Gastroesophageal reflux disease (heartburn, acid regurgitation), epigastric/chest pain, dysphagia, food impaction, vomiting, weight loss	Mucosal form: vomiting, abdominal pain, diarrhea malabsorption, protein‐losing enteropathy, iron‐deficient anemia, failure to thrive (children), melena Muscolaris layer form: obstructive symptoms Serosal form: eosinophil‐rich ascites	Abdominal pain Diarrhea Weight loss Anorexia
Endoscopic features	Edema Linear oriented creases (furrowing) Mucosal rings (feline esophagus) Exudates and whitish papules Polyps Strictures	Micronodules Erosion Mucosal hyperemia	Erythema Loss of vascularity Lymphonodular hyperplasia
Diagnostic criteria	≥15 Eo/HPF from at least one site (distal, mid, or proximal esophagus)	≥30 Eo/HPF in ≥5 HPF or ≥70 Eo/HPF in ≥3 HPF (stomach) ≥52 Eo/HPF (duodenum) ≥56 Eo/HPF (ileum)	≥100 Eo/HPF (cecum/ascending colon) ≥84 Eo/HPF (transverse/descending colon) ≥64 Eo/HPF (sigma/rectum)
Histopathological features	Eosinophilic inflammation, eosinophil abscess, eosinophil surface layer, basal zone hyperplasia, dilated intercellular spaces, dyskeratotic epithelial cells, lamina propria fibrosis. Immunostaining for MCP, ECP, IgE, tryptase	Eosinophilic inflammation in different layers Blunt villi Immunostaining for MCP, ECP, IgE, tryptase	Eosinophil cryptitis/crypt abscesses, crypt architectural abnormalities, increased intraepithelial eosinophils, and eosinophils in muscularis mucosa and submucosa Immunostaining for MCP, ECP, IgE, tryptase
Laboratory parameters	Peripheral blood eosinophilia (not always present)	Peripheral blood eosinophilia (not always present)	Peripheral blood eosinophilia (not always present)
Differential diagnoses	Infection HES Neoplasm CTD/SS Small vessel vasculitis Drug reaction	Infection HES Celiac disease Crohn's disease CTD/SS Small vessel vasculitis Systemic mastocytosis Drug reaction	Infection HES Ulcerative colitis Crohn's disease CTD/SS Small vessel vasculitis Systemic mastocytosis Drug reaction
Association with allergic disorders	+++	++	+
Predominant allergic phenotype	IgE	IgE	T‐cell
	T‐cell	T‐cell	
Therapeutic options	Elemental diet, 6, 4, and 2 FED Topical glucocorticoid Proton pump inhibitors	Elemental diets Topical and systemic glucocorticoid	Elemental diet Topical and systemic glucocorticoid
Evolution	Esophageal stenosis, bleeding, perforation/rupture, especially if left untreated	Poorly characterized in the long term	Poorly characterized in the long term

Abbreviations: CTD, connective tissue disease; FED, food elimination diet; HES, hyper‐eosinophilic syndrome; HPF, high power field; SS, systemic sclerosis.

The aim of the present review is to provide in a narrative and concise fashion an updated overview about the association between EGID and the whole spectrum of allergic disorders in adults and children, in order to improve diagnosis and treatment of allergic comorbidities in patients with EGID. We also provide a critical update of the ongoing clinical trials regarding therapies for EGID, highlighting potential advantages for concomitant allergic disorders.

## 2 METHODS

In June and September 2021, we performed a computer‐assisted literature search for relevant studies using PubMed. The aim of the search was to find papers dealing with the association of EGID with allergic disorders, focusing on the clinical and therapeutical implications. The research was restricted to papers published in English. The medical subject heading terms used were “EoE,” “eosinophilic gastritis,” “eosinophilic gastroenteritis,” “eosinophilic colitis,” and “atopy,” “asthma,” “allergic rhinitis,” “atopic dermatitis,” “drug allergy,” “eczema,” “environmental allergy.” By using these terms, we found more 3000 papers. Of these, most were unrelated to the review topic and hence were discarded by all authors. We focused on the original, review articles, and case reports/series since database inception, dealing with the association of allergic disorders in EGID, in both the pediatric and the adult settings. We also searched for relevant papers cited in authoritative reviews dealing with EGID in relation to other allergic disorders. Given the narrative, expert‐based, nature of the review we did not carry out a systematic review of the literature.

### 2.1 Eosinophilic esophagitis

Eosinophilic esophagitis has proteiform manifestations and symptoms, which vary with age.^4^ While young children and toddlers usually experience vomiting, regurgitation, abdominal pain, feeding refusal, and failure to thrive, adolescents and adults often report dysphagia and food impaction that may be the expression of advanced tissue remodeling.^22^, ^23^, ^24^ EoE may affect people of any age and gender, but it is more common in young male individuals. It is characterized by the presence of esophageal infiltration in both the proximal and distal esophagus. The disrupted function of the muscolaris mucosa layer, which can be shown by ultrasonography, results in symptoms of esophageal dysmotility.^25^

Most of the studies considering the relationship between EGID and asthma are focused on EoE, probably because EoE is the most frequent form of EGID, paralleling the epidemiologic surge of allergic diseases.^7^, ^26^ Several studies have shown that patients with EoE suffer from a significant burden of allergic comorbidities, such allergic rhinitis, asthma, atopic dermatitis, and IgE‐mediated food‐allergy.

The prevalence of asthma in adult series of EoE patients varies from 25% to 50%, and reaches 60% in pediatric series.^26^, ^27^, ^28^ Moreover, in a previous meta‐analysis considering a large number of individuals it was found that patients with EoE had a significantly increased probability of having asthma (OR 3.01, 95% CI 1.96–4.62, OR 5.09, 95% CI 3.91–8.90, respectively) and allergic rhinitis compared to controls.^29^ This strong association has led some authors to consider EoE as “the asthma of the esophagus.”^30^

Food allergy has been traditionally linked to EoE, given the strong epidemiologic link between these disorders, the clinical and histological response of EoE to elemental diets, and, more recently, the increased recognition of EoE in patients being treated with oral immunotherapy.^8^ The prevalence of IgE‐mediated food allergy varies between 25% and nearly 70%.^29^, ^31^ The most frequently implied foods are milk, wheat, soy, egg, nuts, and shellfish. Eczema was also significantly more frequent in patients than controls, (OR 2.85, 95% CI 1.87–4.34).

Finally, in a large cross‐sectional, population‐based survey conducted in the US, a high prevalence of allergic disorders was observed among 74 EoE children and 89 EoE adults, namely 32.4% and 37.3%, respectively, had ≥1 current IgE‐food allergy, 27.8% and 47.8%, respectively, had asthma, 27.5% and 22.9%, respectively, had atopic dermatitis/eczema, and 43.5% and 41.6%, respectively, had seasonal rhinitis.^9^

Overall, these findings have led many researchers to include EoE in the spectrum of disorders making up the atopic march, often representing the final step of this progression.^32^ Of note, the association between food allergy and EoE has been found to be the strongest.^32^

Several pathophysiological theories have been put forward to explain the association between EoE with atopic disorders, however a consistent picture is still lacking.^33^ A possible role of aeroallergens in terms of EoE diagnosis/exacerbation has been suggested by clinical studies, showing an association between pollen season and incidence of EoE diagnosis.^34^ Besides, cases of EoE after sublingual immunotherapy for respiratory allergies have also been observed.^20^, ^35^, ^36^ The exact mechanistic interpretation of these findings is still incomplete. A direct effect of pollen allergens, but also of food allergens that are cross‐reactive to pollens, could be present.

A common pathophysiologic feature of EoE and food allergy could be the presence of a shared allergen‐restricted Th2 specificity. However, despite these similarities, these conditions display peculiar features, as EoE is usually a life‐long disease, whereas food allergy is usually transitory, so it is not uncommon to encounter patients with EoE with a history of food allergy. Moreover, the anti‐IgE therapy seems to exert a marginal role in EoE.^37^ ^,^ ^38^ These findings imply that the eosinophilic inflammation in EoE is independent of a classical Th2‐response and other still unknown factors play a role.

### 2.2 Eosinophilic gastritis and gastroenteritis

Gastritis, enteritis, and gastroenteritis are usually considered as a whole nosologic entity given their clinical similarities and paucity of pathogenetic knowledge. They may show concomitant eosinophilic infiltration of other gut regions, such as the esophagus and the large intestine. Clinical manifestations are proteiform, as already shown in Table 1, depending on which layer of the gut wall is mostly affected. Symptoms could be mild and often overlooked, or could be serious and potentially life‐threatening, including abdominal pain, diarrhea, and frank malabsorption.^39^

Asthma and other allergic diseases, such as allergic rhinitis, have also been described in patients with eosinophilic gastritis or gastroenteritis, but with less convincing evidence compared to EoE. Nonetheless, the frequency of self‐reported allergic rhinitis and asthma is still relevant, as high as 63% and 39%, respectively, in a questionnaire‐based registry study assessing the prevalence of atopic conditions in 107 patients, adults and children, with these conditions.^40^

More recently, some case reports have described the association between asthma and eosinophilic gastritis in a few patients with severe asthma, treated with dupilumab or mepolizumab.^41^ ^,^ ^42^ Few data pertaining the association between eosinophilic gastritis with food allergy are available, while the majority of the studies has evaluated mainly sensitization to food allergens alone. Another limitation is represented by the inclusion of cases of concomitant EoE. The presence of food allergy was ascertained in a pediatric US series in one‐ninth of patients with isolated eosinophilic gastritis and one‐third in those with eosinophilic gastritis with duodenal eosinophilia.^43^ in an another US study including 44 patients, children and adults, with eosinophilic gastroenteritis (associated EoE in 30% of the cases) the prevalence of food allergy was 42%. Interestingly, drug allergy was also found in 31% and eczema in 16%.^44^

Overall, the prevalence of atopic disorders in patients with eosinophilic gastritis and gastroenteritis appears to be high, being estimated at 38.5% and 45.6%, respectively.^10^

### 2.3 Eosinophilic colitis

Primary eosinophilic colitis is the least frequent disorder among EGID. The absence of internationally agreed diagnostic criteria, including a clear eosinophilic infiltrate threshold, has hampered its identification for a long time. Eosinophilic colitis frequently presents with diarrhea, abdominal pain, anorexia, and weight loss. It has a bimodal age presentation, namely in infancy (at approximately 60 days of age) and during adolescence and early adulthood.^45^ Also, it has been associated with a wide range of atopic disorders, including drug allergy, allergic rhinitis, asthma, and food allergy.^46^ ^,^ ^47^

In a US administrative database study, Jensen et al. evaluated 404 adult patients with eosinophilic colitis, finding that co‐existing allergic conditions were common, being present in 41.8% of the patients.^10^ The most commonly reported allergic condition was allergic rhinitis (30%). Asthma was reported in 15% and atopic dermatitis in 6.2% of the patients. In a smaller series of adult patients (*n* = 22), a lower incidence of both asthma and allergic rhinitis (18%) was reported.^47^

The prevalence of atopic conditions seems to be high also in children, according to the only case series available, which includes almost 50 individuals, and reports that 40% displayed one or more signs of atopy.^48^ The same estimate of comorbid atopic conditions has been calculated by Dellon et al. in the aforementioned register‐based study.^10^

## 3 OUTLOOK

Allergic manifestations are a frequent comorbidity in patients with immune‐mediated disorders of the gastrointestinal tract, including classical autoimmune diseases and EGID.^49^ The current evidence of the association between EGID and allergic disorders, as discussed above, is summarized in Tables 2, 3, 4, for adults, children, and both, respectively.

**TABLE 2:** Studies describing the association between eosinophilic gastrointestinal disorders and allergic disorders in adults

Author, year, country	Study type	Population	Pathology	Allergy disease	Comments	References
Wright et al., 2018 United States	Prospective Patients with peanut allergy (n = 21)	Adults	EoE	Food allergy	EoE common in adults with IgE‐mediated peanut allergy before OIT	50
Eckmann et al., 2018 United States	Pilot, prospective, open‐label. EoE (*n* = 8)	Adults	EoE	Food allergy	Only four patients with a positive atopy patch test. No concordance between atopy patch test and EoE	51
Burk et al., 2017 United States	Prospective EoE patients with peanut allergy (*n* = 13)	Adults	EoE	Food allergy	Two patients pretreated with omalizumab developed EoE	52
Dellon et al., 2015 United States	Prospective, case‐control EoE (n = 81), controls (n = 144)	Adults	EoE	Asthma Rhinitis Atopic dermatitis Food allergy	Food allergies more common in EoE while atopy disease had not statistical significance	27
Sealock et al., 2013 United States	Prospective, case‐control. EoE patients (*n* = 31), esophageal eosinophilia without EoE (*n* = 7), controls (*n* = 1319)	Adults	EoE	Asthma	Seasonal allergies and esophageal strictures associated with esophageal eosinophilia. Asthma not significantly associated with esophageal eosinophilia or EoE	53
Joo et al., 2012 Korea	Prospective. EoE patients(*n* = 8) and controls (*n* = 114)	Adults	EoE	Asthma Rhinitis Atopic dermatitis Food allergy	A history of allergic rhinitis and atopic dermatitis significantly common in EoE patients	54
DeBrosse et al., 2011 Unites States	Retrospective, nested case‐control. EoE patients (*n* = 42), chronic esophagitis (*n* = 67), controls (*n* = 100)	Adults	EoE	Rhinitis Food allergy	Food impaction more common in patients with food allergy. Eczema associated with history of esophageal dilation Allergic rhinitis, asthma, and food allergy associate with dysphagia Food allergy more frequent in EoE patients than chronic esophagitis	55
Garcia‐Compean et al., 2011 Mexico	Prospective. EoE patients (*n* = 6) and controls (*n* = 144)	Adults	EoE	Atopy	Atopy as an independent predictor of EoE	56
Ravi et al., 2011 United States	Retrospective. EoE patients (*n* = 418) and controls (*n* = 59)	Adults	EoE	Asthma Rhinitis	Atopy (asthma and allergic rhinitis) more common in patients with ≥15 eos/HPF	57
Foroutan et al., 2010 Iran	Cross‐sectional. EoE patients (*n* = 6), controls (*n* = 62)	Adults	EoE	Asthma Rhinitis Atopic dermatitis Food allergy Atopy	Atopy was more common in EoE, while asthma, urticaria, atopic dermatitis, rhino‐conjunctivitis, and food allergy had not significant values	58
Veerappan et al., 2009 United States	Prospective. EoE patients (*n* = 25) controls (*n* = 360)	Adults	EoE	Asthma	Higher asthma rates in patients with EoE than controls	59
Mackenzie et al., 2008 United States	Prospective. EoE patients (*n* = 31) and non‐EoE patients (*n* = 230)	Adults	EoE	Asthma Food allergy	12% of patients had EoE pathological criteria. EoE more common in patients with asthma and self‐reported food allergy	60

Abbreviations: EoE, eosinophilic esophagitis; GERD, gastroesophageal reflux disease; OIT, oral immunotherapy.

**TABLE 3:** Studies describing the association between eosinophilic gastrointestinal disorders and allergic disorders in children

Author, year, country	Study type	Population	Pathology	Allergy disease	Comments	References
Votto et al., 2021 Italy	Retrospective	Children and adolescents	EGIDs	Asthma Rhinitis Atopic dermatitis Food allergy	Allergic comorbidities in approximately 30% of enrolled patients, more frequently observed in children with EoE (36.5%)	61
Leung et al., 2015 Canada	Prospective. EoE (n = 23), GERD (n = 20), normal superior endoscopy with gastrointestinal symptoms (n = 14) and controls (n = 26)	Children	EoE	Asthma Rhinitis Atopic dermatitis Food allergy	Rhinitis more common in EoE group	62
Fuentes‐Aparicio et al., 2013 Spain	Randomized clinical trial. Patients with egg allergy (*n* = 40)	Children	EoE	Food allergy	One patient developed EoE after egg OIT	63
Slae et al., 2013 Canada	Cross‐sectional, case‐control study. EoE patients (*n* = 102) and controls (*n* = 167)	Children	EoE	Asthma Rhinitis Atopic dermatitis Food allergy	Food allergy, (peanuts and tree nuts) allergy to pollen (tree and grass) significantly higher among EoE than controls	64
Jensen et al., 2013 United States	Case‐control. EoE patients (*n* = 31), GERD (*n* = 26), and siblings of non‐syndromic cleft lip/palate patients (*n* = 26)	Children	EoE	Asthma Food allergy	The frequency of food allergies, environmental allergies, and asthma higher for cases with EoE than controls	65
Sanchez‐Garcia et al., 2012 Spain	Retrospective. Patients with milk allergy (*n* = 110)	Children	EoE	Food allergy	Three patients developed EoE after milk OIT	66
Ridolo et al., 2011 Italy	Case report	Children	EoE	Food allergy	A child with acute EoE after egg OIT	67
Cassel et al., 2009 United States	Retrospective charts review. EoE patients (*n* = 35) and controls (*n* = 7)	Children	EoE	Asthma Atopic dermatitis	Atopy, asthma, and eczema were more common in EoE patient than in GERD	68
Aceves et al., 2009 United States	Prospective, case‐control. Patients with EoE (*n* = 35), GERD (*n* = 27), allergic patients without EoE (*n* = 24), and non‐allergic patients (*n* = 14)	Children	EoE	Asthma Rhinitis Food allergy	Food allergy more common in patients with EoE while asthma and allergic rhinitis were more common in allergic controls	69
Hofmann et al., 2009 United States	Retrospective. Patients undergoing oral immunotherapy (OIT) (*n* = 28)	Children	EoE	Food allergy	One patient developed EoE after OIT	70
Sugnaman et al., 2007 Australia	Prospective. EoE patients (*n* = 45) and controls (*n* = 33)	Children	EoE	Asthma Rhinitis Atopic dermatitis Anaphylaxis	Atopic eczema, asthma, and rhinitis significantly increased in EoE patients than controls. Incidence of anaphylaxis 24%	71

Abbreviations: EoE, eosinophilic esophagitis; GERD, gastroesophageal reflux disease; OIT, oral immunotherapy.

**TABLE 4:** Studies describing the association between eosinophilic gastrointestinal disorders and allergic disorders in both adults and children

Author, year, country	Study type	Population	Pathology	Allergy disease	Comments	References
Duffey et al., 2016 United States	Retrospective, administrative data. EoE and their relatives (*n* = 4.009) and controls (*n* > 100.000)	Children and adults	EoE	Asthma	Significant familial clustering of asthma and atopic disease (anaphylaxis, atopic dermatitis, allergic rhinitis, and conjunctivitis) in distant relatives of EoE proband	72
Peterson et al., 2015 United States	Retrospective, case‐control. EoE (n = 4423) and controls (first‐ and second‐degree relatives, first cousin and spouses of patients) (n = 22.627)	Children and adults	EoE	Asthma Rhinitis Atopic dermatitis Food allergy Anaphylaxes	Atopy diseases including anaphylaxes more common in EoE patients and relatives	73
Mansoor et al., 2016 United States	Administrative data. EoE patients (*n* = 7840), whole population controls (*n* = 30,301,440)	Children and adults	EoE	Asthma Rhinitis Atopic dermatitis Food allergy Drug allergy	Allergic diseases (drug allergy, food allergy, rhinitis, IgE mediated disorder, asthma, sinusitis, dermatitis, eczema, and urticaria) more common in EoE patients	74
Mulder et al., 2013 Canada	Retrospective, case‐control. EoE patients (*n* = 44) and controls (*n* = 44)	Children and adults	EoE	Asthma Rhinitis Atopic dermatitis Food allergy Drug allergy	Atopy more common in EoE patients than controls	81
Zafra et al., 2013 Spain	Prospective, case‐control. EoE (*n* = 25) and controls (*n* = 17)	Children and adults	EoE	Rhinitis Food allergy	EoE patients more likely to have sensibilization to aeroallergens, rhino conjunctivitis, and food allergy	75
Dellon et al., 2009 United States	Retrospective case‐control. EoE patients (*n* = 151), GERD (*n* = 226)	Children and adults	EoE	Asthma Rhinitis Atopic dermatitis Food allergy	Atopy (allergic rhinitis/dermatitis, food allergy, and asthma) was more common in EoE patients and food allergy was considered a reliably predictor factor to discriminate EoE from GERD	76

Abbreviations: EoE, eosinophilic esophagitis; GERD, gastroesophageal reflux disease.

Allergens can lead to disease exacerbation and allergen elimination results in disease control in a significant proportion of patients. Besides, the control of atopic conditions is important to control EoE.^77^ Patients living with EGID should be carefully evaluated from a multidisciplinary team, made up by an allergist, a pediatrician, and a gastroenterologist, considering all aspects of Th2 inflammation. Treatment modalities should possibly be tailored to tackle shared molecular pathways.

**FIGURE 1:**
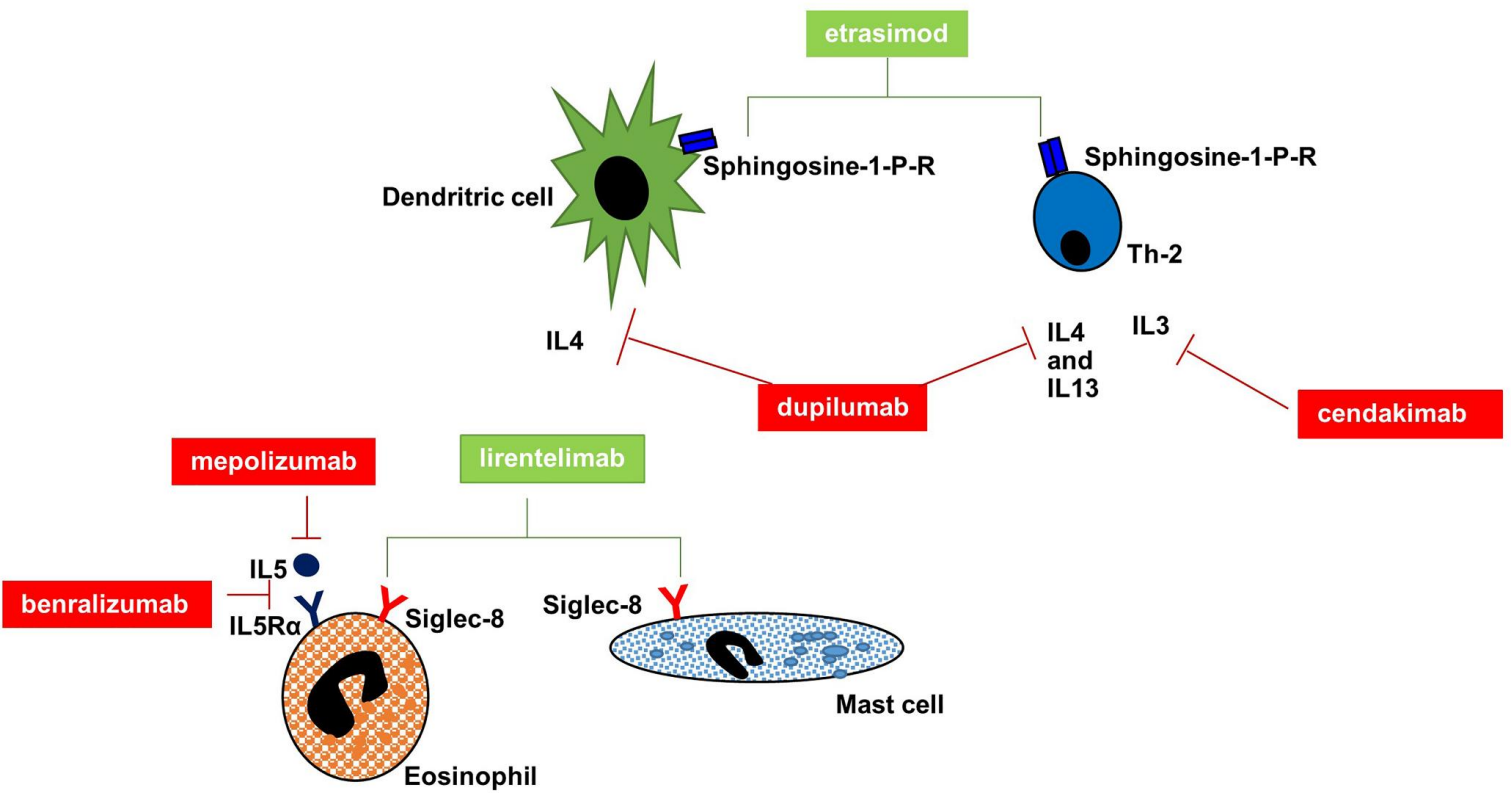
Main molecular targets of monoclonal antibodies in primary eosinophilic gastrointestinal disorders (EGID). Monoclonal antibodies (mAbs) available in clinical practice for other Th2 disorders and under evaluation in clinical trials in EGID are shown along with the main cellular effectors of Th2 response (i.e., dendritic cells, mast cells, Th2 cells, and eosinophils). The red lines denote an inhibitory action, such as for mAbs against interleukin (IL)5 (mepolizumab) or IL5 receptor (benzalizumab), IL4/IL13 (dupilumab), and IL3 (cendakimab), whereas the green lines denote a modulatory effect, such as for etrasimob on sphingosine 1‐P receptor and lirentelimab on Siglec‐8

Notably, a number of clinical trials regarding treatment modalities for EGID are currently ongoing (Table 5). Apart from a few unspecific, non‐biologic, molecules, most of the drugs under investigations are monoclonal antibodies, all of them targeting different pathogenic pathways that, in some cases, are shared with allergic diseases. For example, dupilumab, an anti‐interleukin 4 (IL4) receptor alpha monoclonal antibody, has already been approved for the treatment of atopic dermatitis and allergic asthma, while mepolizumab, an anti‐IL5 monoclonal antibody, has already been approved for allergic asthma.^78^ ^,^ ^79^ Moreover, lirentelimab, a monoclonal antibody targeting an inhibitory receptor Siglec‐8, could represent an interesting therapeutical agent targeting both the allergic disorders and EGID, since this receptor is present only on mastcells, basophils, and eosinophils, all key players in both disease groups.^18^, ^80^, ^81^ The main molecular targets of monoclonal antibodies are shown in Figure 1.

**TABLE 5:** The currently ongoing clinical trials for the treatment of primary eosinophilic gastrointestinal disorders

Agent	Route of administration	Mechanism of action	Condition	Clinical trial number	Phase
Antihistamines (loratadine and famotidine)	Oral	Histamine‐1 (H1) and Histamine‐2 (H2) receptor antagonists	EoE	NCT04248712	2
Febuxostat	Oral	Non‐purine‐selective inhibitor of xanthine oxidase	EoE	NCT02873468	2
Omeprazole	Oral	PPI	EoE	NCT04149470	4
Fluticasone and omeprazole versus fluticasone alone	Oral	Anti‐inflammatory PPI	EoE	NCT03781596	4
Budesonide	Oral	Anti‐inflammatory	EoE	NCT03245840	3
Fluticasone propionate	Oral	Anti‐inflammatory	EoE	NCT04281108	3
Mometasone furoate	Oral	Anti‐inflammatory	EoE	NCT04849390	2
Mepolizumab	s.c.	Anti‐IL5 mAb	EoE	NCT03656380	2
Benralizumab	s.c.	Anti‐IL5Rα mAb	EoG	NCT03473977	2–3
			EoGE		
Benralizumab	s.c.	Anti‐IL5Rα mAb	EoE	NCT04543409	3
Dupilumab	s.c.	Anti‐IL4/13 mAb	EoG	NCT03678545	2
			EoGE		
Dupilumab	s.c.	Anti‐IL4/13 mAb	EoE	NCT03633617	3
Dupilumab	s.c.	Anti‐IL4/13 mAb	EoE	NCT04394351	3
Cendakimab	s.c.	Anti‐IL3 mAb	EoE	NCT04753697	3
CALY‐002	i.v.	Anti‐IL15 mAb	EoE	NCT04593251	1
			Celiac disease		
Lirentelimab	i.v.	Anti‐Siglec‐8 mAb	EoE	NCT04322708	2–3
Lirentelimab	i.v.	Anti‐Siglec‐8 mAb	EoG	NCT04322604	3
			EoGE		
			EoD		
Lirentelimab	i.v.	Anti‐Siglec‐8 mAb	EoG	NCT03664960	2
			EoGE		
			EoD		
Lirentelimab	i.v	Anti‐Siglec‐8 mAb	EoG	NCT04620811	3
			EoGE		
			EoD		
Lirentelimab	i.v.	Anti‐Siglec‐8 mAb	EoGE	NCT04856891	3
			EoD		
Etrasimod	Oral	Sphingosine 1‐phosphate (S1P) receptor	EoE	NCT04682639	2
Benzimidazolylpicolinoyl	Oral	Active lanthionine synthetase C‐like 2 (LANCL2)	EoE	NCT04835168	1

Abbreviations: EoD, eosinophilic duodenitis; EoE, eosinophilic esophagitis; EoG, eosinophilic gastritis; EoGE, eosinophilic gastroenteritis; mAb, monoclonal antibody; PPI, proton pump inhibitor.

We do feel that EGID and allergic disorders should be better managed by a multidisciplinary team, given their complex nature, which is not only confined to their possible shared pathophysiological bases, but also includes (i) the high clinical burden, due to their potentially long diagnostic delay and poor quality of life, (ii) the difficult diagnostic work‐up, and (iii) the need for specific expertise and competences for their diagnosis. The future clinical research agenda should focus on the identification of non‐invasive biomarkers for their diagnosis and their early recognition. The main key messages mentioned in the outlook are summarized in Table 6.^82^

**TABLE 6:** Key messages

A multidisciplinary approach for the diagnosis and treatment of EGID is warranted to tackle all the diverse organ manifestations of Th2 inflammation (i.e., skin, nose, and lungs, gastrointestinal tract).
2. The identification of the causal allergen(s) improves disease control.
3. Pathogenesis‐targeted therapies aimed at controlling the whole burden of allergic comorbidities within the same patient should be considered.
4. Non‐invasive diagnostic biomarkers to enable early diagnosis are needed.

## CONFLICT OF INTEREST

None to disclose for all authors.

## AUTHOR CONTRIBUTIONS

All authors significantly participated in the drafting of the manuscript or critical revision of the manuscript for important intellectual content and provided approval of the final submitted version.

## References

[1] Cianferoni A , Spergel J . Eosinophilic esophagitis and gastroenteritis. Curr Allergy Asthma Rep. 2015;15:58. 10.1007/S11882-015-0558-5 26233430

[2] Spergel J , Brown‐Whitehorn T , Muir A , et al. Medical algorithm: diagnosis and treatment of eosinophilic esophagitis in children. Allergy. 2020;75:1522‐1524. 31953959 10.1111/all.14188 PMC8040021

[3] Pineton de Chambrun G , Gonzalez F , Canva J , et al. Natural history of eosinophilic gastroenteritis. Clin Gastroenterol Hepatol. 2011;9:950‐956. 21806952 10.1016/j.cgh.2011.07.017

[4] Straumann A , Aceves S , Blanchard C , et al. Pediatric and adult eosinophilic esophagitis: similarities and differences. Allergy. 2012;67:477‐490. 22313241 10.1111/j.1398-9995.2012.02787.x

[5] Gonsalves N . Eosinophilic gastrointestinal disorders. Clin Rev Allergy Immunol. 2019;57:272‐285. 30903439 10.1007/s12016-019-08732-1

[6] Alhmoud T , Hanson J , Parasher G . Eosinophilic gastroenteritis: an underdiagnosed condition. Dig Dis Sci. 2016;61:2585‐2592. 27234270 10.1007/s10620-016-4203-5

[7] Dellon ES , Hirano I . Epidemiology and natural history of eosinophilic esophagitis. Gastroenterology. 2018;154:319‐332. 28774845 10.1053/j.gastro.2017.06.067 PMC5794619

[8] Capucilli P , Hill DA . Allergic comorbidity in eosinophilic esophagitis: mechanistic relevance and clinical implications. Clin Rev Allergy Immunol. 2019;57:111‐127. 30903437 10.1007/s12016-019-08733-0 PMC6626558

[9] Cianferoni A , Warren CM , Brown‐Whitehorn T , et al. Eosinophilic esophagitis and allergic comorbidities in a US‐population‐based study. Allergy. 2020;75:1466‐1469. 31846078 10.1111/all.14148 PMC8020642

[10] Jensen ET , Martin CF , Kappelman MD , et al. Prevalence of eosinophilic gastritis, gastroenteritis, and colitis: estimates from a national administrative database. J Pediatr Gastroenterol Nutr. 2016;62:36‐42. 25988554 10.1097/MPG.0000000000000865 PMC4654708

[11] Lenti M , Corazza G . Administrative data for exploring multimorbidity in hospitalised patients. Intern Emerg Med. 2020;15:1161‐1163. 32193771 10.1007/s11739-020-02307-1

[12] Licari A , Votto M , Scudeller L , et al. Epidemiology of nonesophageal eosinophilic gastrointestinal diseases in symptomatic patients: a systematic review and meta‐analysis. J Allergy Clin Immunol Pract. 2020;8:1994‐2003. 32061717 10.1016/j.jaip.2020.01.060

[13] Dellon E , Liacouras C , Molina‐Infante J , et al. Updated international consensus diagnostic criteria for eosinophilic esophagitis: proceedings of the AGREE conference. Gastroenterology. 2018;155:1022‐1033. 30009819 10.1053/j.gastro.2018.07.009 PMC6174113

[14] Votto M , Marseglia G , De Filippo M , et al. Early life risk factors in pediatric EoE: could we prevent this modern disease? Front Pediatr. 2020;8:263. 32548083 10.3389/fped.2020.00263 PMC7274037

[15] Caldwell J , Collins M , Stucke E , et al. Histologic eosinophilic gastritis is a systemic disorder associated with blood and extragastric eosinophilia, TH2 immunity, and a unique gastric transcriptome. J Allergy Clin Immunol. 2014;134:1114‐1124. 25234644 10.1016/j.jaci.2014.07.026 PMC4254306

[16] Talley N . Gut eosinophilia in food allergy and systemic and autoimmune diseases. Gastroenterol Clin North Am. 2008;37:307‐332. 18499022 10.1016/j.gtc.2008.02.008

[17] Lenti MV , Savarino E , Mauro A , et al. Diagnostic delay and misdiagnosis in eosinophilic oesophagitis. Dig Liver Dis. 2021;53(12):1632‐1639. 10.1016/J.DLD.2021.05.017 34116974

[18] Peterson K , Safroneeva E , Schoepfer A . Emerging therapies for eosinophilic gastrointestinal diseases. J Allergy Clin Immunol Pract. 2021;9:3276‐3281. 34343695 10.1016/j.jaip.2021.07.031

[19] Afinogenova Y , Rubin T , Patel S , et al. Community private practice clinical experience with peanut oral immunotherapy. J Allergy Clin Immunol Pract. 2020;8:2727‐2735. 32247684 10.1016/j.jaip.2020.03.016

[20] Antico A , Fante R . Esophageal hypereosinophilia induced by grass sublingual immunotherapy. J Allergy Clin Immunol. 2014;133:1482‐1484. 24636095 10.1016/j.jaci.2014.01.030

[21] Collins M , Capocelli K , Yang G . Eosinophilic gastrointestinal disorders pathology. Front Med. 2018;4:261. 10.3389/fmed.2017.00261 PMC5775510 29379785

[22] Schoepfer A , Safroneeva E , Bussmann C , et al. Delay in diagnosis of eosinophilic esophagitis increases risk for stricture formation in a time‐dependent manner. Gastroenterology. 2013;145:1230‐1236. 23954315 10.1053/j.gastro.2013.08.015

[23] Muir A , Brown‐Whitehorn T , Godwin B , et al. Eosinophilic esophagitis: early diagnosis is the key. Clin Exp Gastroenterol. 2019;12:391‐399. 31616174 10.2147/CEG.S175061 PMC6699505

[24] Votto M , Castagnoli R , De Filippo M , et al. Behavioral issues and quality of life in children with eosinophilic esophagitis. Minerva Pediatr. 2020;72:424‐432. 32506880 10.23736/S0026-4946.20.05913-7

[25] Clarke J , Ahuja N , Fernandez‐Becker N , et al. The functional lumen imaging probe in gastrointestinal disorders: the past, present, and future. Ann N Y Acad Sci. 2020;1482:16‐25. 32814368 10.1111/nyas.14463

[26] Capucilli P , Cianferoni A , Grundmeier RW , et al. Comparison of comorbid diagnoses in children with and without eosinophilic esophagitis in a large population. Ann Allergy Asthma Immunol. 2018;121(6):711‐716. 30194971 10.1016/j.anai.2018.08.022

[27] Dellon ES , Rusin S , Gebhart JH , et al. A clinical prediction tool identifies cases of eosinophilic esophagitis without endoscopic biopsy: a prospective study. Am J Gastroenterol. 2015;110(9):1347‐1354. 26303128 10.1038/ajg.2015.239 PMC4586067

[28] Chehade M , Jones SM , Pesek RD , et al. Phenotypic characterization of eosinophilic esophagitis in a large multicenter patient population from the consortium for food allergy research. J Allergy Clin Immunol Pract. 2018;6(5):1534‐1544. 30075341 10.1016/j.jaip.2018.05.038 PMC6132253

[29] González‐Cervera J , Arias Á , Redondo‐González O , et al. Association between atopic manifestations and eosinophilic esophagitis a systematic review and meta‐analysis. Ann Allergy Asthma Immunol. 2017;118(5):582‐590. 28366582 10.1016/j.anai.2017.02.006

[30] Virchow J . Eosinophilic esophagitis: asthma of the esophagus? Dig Dis. 2014;32:54‐60. 24603381 10.1159/000357010

[31] Hill DA , Dudley JW , Spergel JM . The prevalence of eosinophilic esophagitis in pediatric patients with IgE‐mediated food allergy. J Allergy Clin Immunol Pract. 2017;5:369‐375. 28042003 10.1016/j.jaip.2016.11.020 PMC5346349

[32] Hill DA , Grundmeier RW , Ramos M , et al. Eosinophilic esophagitis is a late manifestation of the allergic march. J Allergy Clin Immunol Pract. 2018;6(5):1528‐1533. 29954692 10.1016/j.jaip.2018.05.010 PMC6131029

[33] Ruffner M , Cianferoni A . Phenotypes and endotypes in eosinophilic esophagitis. Ann Allergy Asthma Immunol. 2020;124:233‐239. 31862435 10.1016/j.anai.2019.12.011

[34] Mishra A , Hogan SP , Brandt EB , et al. An etiological role for aeroallergens and eosinophils in experimental esophagitis. J Clin Invest. 2001;107:83‐90. 11134183 10.1172/JCI10224 PMC198543

[35] Wells R , Fox A , Furman M . Recurrence of eosinophilic oesophagitis with subcutaneous grass pollen immunotherapy. BMJ Case Rep. 2018;2018:bcr2017223465. 10.1136/BCR-2017-223465 PMC5878312 29545433

[36] Béné J , Ley D , Roboubi R , et al. Eosinophilic esophagitis after desensitization to dust mites with sublingual immunotherapy. Ann Allergy Asthma Immunol. 2016;116:583‐584. 27067457 10.1016/j.anai.2016.03.017

[37] Loizou D , Enav B , Komlodi‐Pasztor E , et al. A pilot study of omalizumab in eosinophilic esophagitis. PLoS One. 2015;10(3):e0113483. 25789989 10.1371/journal.pone.0113483 PMC4366078

[38] Clayton F , Fang JC , Gleich GJ , et al. Eosinophilic esophagitis in adults is associated with IgG4 and not mediated by IgE. Gastroenterology. 2014;147:602‐609. 24907494 10.1053/j.gastro.2014.05.036

[39] Pesek R , Greuter T , Lopez‐Nunez O , et al. Clinicopathologic correlations in eosinophilic gastrointestinal disorders. J Allergy Clin Immunol Pract. 2021;9:3258‐3266. 34507707 10.1016/j.jaip.2021.06.002

[40] Guajardo JR , Plotnick LM , Fende JM , et al. Eosinophil‐associated gastrointestinal disorders: a world‐wide‐web based registry. J Pediatr. 2002;141(4):576‐581. 12378201 10.1067/mpd.2002.127663

[41] Iwamuro M , Murakami T , Tanaka T , et al. Eosinophilic gastritis in a patient previously treated with dupilumab. Case Rep Gastrointest Med. 2020;2020:6381670. 10.1155/2020/6381670 32566328 PMC7292988

[42] Han D , Lee JK . Severe asthma with eosinophilic gastroenteritis effectively managed by mepolizumab and omalizumab. Ann Allergy Asthma Immunol. 2018;121(6):742‐743. 30056148 10.1016/j.anai.2018.07.030

[43] Ko H , Morotti R , Yershov O , et al. Eosinophilic gastritis in children: clinicopathological correlation, disease course, and response to therapy. Am J Gastroenterol. 2014;109:1277‐1285. 24957155 10.1038/ajg.2014.166

[44] Reed C , Woosley JT , Dellon ES . Clinical characteristics, treatment outcomes, and resource utilization in children and adults with eosinophilic gastroenteritis. Dig Liver Dis. 2015;47:197. 25547198 10.1016/j.dld.2014.11.009 PMC4339627

[45] Raffaele A , Vatta F , Votto M , et al. Eosinophilic colitis in children: a new and elusive enemy? Pediatr Surg Int. 2021;37:485‐490. 33409540 10.1007/s00383-020-04832-8

[46] Impellizzeri G , Marasco G , Eusebi LH , et al. Eosinophilic colitis: a clinical review. Dig Liver Dis. 2019;51:769‐773. 31122823 10.1016/j.dld.2019.04.011

[47] Díaz del Arco C , Taxonera C , Olivares D , et al. Eosinophilic colitis: case series and literature review. Pathol Res Pract. 2018;214:100‐104. 29103770 10.1016/j.prp.2017.09.029

[48] Behjati S , Zilbauer M , Heuschkel R , et al. Defining eosinophilic colitis in children: insights from a retrospective case series. J Pediatr Gastroenterol Nutr. 2009;49:208‐215. 19525875 10.1097/MPG.0b013e31818de373

[49] Rossi C , Lenti M , Merli S , et al. Allergic manifestations in autoimmune gastrointestinal disorders. Autoimmun Rev. 2022;21(1):102958. 10.1016/j.autrev.2021.102958 34560305

[50] Wright BL , Fernandez‐Becker NQ , Kambham N , et al. Baseline gastrointestinal eosinophilia is common in oral immunotherapy subjects with IgE‐mediated peanut allergy. Front Immunol. 2018;9:1‐12. 30524424 10.3389/fimmu.2018.02624 PMC6261984

[51] Eckmann JD , Ravi K , Katzka DA , et al. Efficacy of atopy patch testing in directed dietary therapy of eosinophilic esophagitis: a pilot study. Dig Dis Sci. 2018;63:694‐702. 29349695 10.1007/s10620-018-4928-4

[52] Burk CM , Dellon ES , Steele PH , et al. Eosinophilic esophagitis during peanut oral immunotherapy with omalizumab. J Allergy Clin Immunol Pract. 2017;5:498‐501. 28017628 10.1016/j.jaip.2016.11.010 PMC5346340

[53] Sealock RJ , Kramer JR , Verstovsek G , et al. The prevalence of oesophageal eosinophilia and eosinophilic oesophagitis: a prospective study in unselected patients presenting to endoscopy. Aliment Pharmacol Ther. 2013;37:825‐832. 23441936 10.1111/apt.12268 PMC3602156

[54] Joo MK , Park J , Kim S , et al. Original article prevalence and endoscopic features of eosinophilic esophagitis in patients with esophageal or upper gastrointestinal symptoms. J Dig Dis. 2012;13(6):296‐303. 22624552 10.1111/j.1751-2980.2012.00589.x

[55] Debrosse CW , Franciosi JP , King EC , et al. Long‐term outcomes in pediatric‐onset esophageal eosinophilia. J Allergy Clin Immunol. 1999;128:132‐138. 10.1016/j.jaci.2011.05.006 PMC3130990 21636117

[56] García‐compeán D , Alberto J , González G , et al. Prevalence of eosinophilic esophagitis in patients with refractory gastroesophageal reflux disease symptoms: a prospective study. Dig Liver Dis. 2011;43:204‐208. 20843755 10.1016/j.dld.2010.08.002

[57] Ravi K , Talley NJ , Smyrk TC , et al. Low grade esophageal eosinophilia in adults: an unrecognized part of the spectrum of eosinophilic esophagitis. Dig Dis Sci. 2011;56(7):1981‐1986. 21298480 10.1007/s10620-011-1594-1

[58] Foroutan M , Norouzi A , Molaei M , et al. Eosinophilic esophagitis in patients with refractory gastroesophageal reflux disease. Dig Dis Sci. 2010;55:28‐31. 19241170 10.1007/s10620-008-0706-z

[59] Veerappan GR , Perry JL , Duncan TJ , et al. Prevalence of eosinophilic esophagitis in an adult population undergoing upper endoscopy: a prospective study. Clin Gastroenterol Hepatol. 2009;7:420‐426. 19162236 10.1016/j.cgh.2008.10.009

[60] Mackenzie SH , Go M , Chadwick B , et al. Eosinophilic oesophagitis in patients presenting with dysphagia ‐ a prospective analysis. Aliment Pharmacol Ther. 2008;28:1140‐1146. 18624788 10.1111/j.1365-2036.2008.03795.x

[61] Votto M , Raffele A , De Filippo M , et al. Eosinophilic gastrointestinal disorders in children and adolescents: a single‐center experience. Dig Liver Dis. 2022;54:214‐220. 34274254 10.1016/j.dld.2021.06.027

[62] Leung AJT , Persad S , Slae M , et al. Intestinal and gastric permeability in children with eosinophilic esophagitis and reflux esophagitis. J Pediatr Gastroenterol Nutr. 2015;60:236‐239. 25304889 10.1097/MPG.0000000000000590

[63] Fuentes‐Aparicio V , Alvarez‐Perea A , Infante S , et al. Specific oral tolerance induction in paediatric patients with persistent egg allergy. Allergol Immunopathol. 2013;41:143‐150. 10.1016/j.aller.2012.02.007 22835606

[64] Slae M , Persad R , Leung AJT , et al. Role of environmental factors in the development of pediatric eosinophilic esophagitis. Dig Dis Sci. 2015;60:3364‐3372. 26062820 10.1007/s10620-015-3740-7

[65] Jensen ET , Kappelman MD , Kim HP , et al. Early life exposures as risk factors for pediatric eosinophilic esophagitis. J Pediatr Gastroenterol Nutr. 2013;57:67‐71. 23518485 10.1097/MPG.0b013e318290d15a

[66] Sánchez‐García S , Rodríguez Del Río P , Escudero C , et al. Possible eosinophilic esophagitis induced by milk oral immunotherapy. J Allergy Clin Immunol. 2012;129:1155‐1157. 22236725 10.1016/j.jaci.2011.11.042

[67] Ridolo E , De Angelis GL , Dall’Aglio P . Eosinophilic esophagitis after specific oral tolerance induction for egg protein. Ann Allergy Asthma Immunol. 2011;106:73‐74. 21195949 10.1016/j.anai.2010.10.010

[68] Cassell H , Mukkada VA , Woodruff SA , et al. Allergic diseases in children with eosinophilic esophagitis (EoE) compared to gastroesophageal reflux disease (GERD). Gastroenterology. 2009;136:284‐285.

[69] Aceves SS , Newbury RO , Dohil MA , Bastian JF , Dohil R . A symptom scoring tool for identifying pediatric patients with eosinophilic esophagitis and correlating symptoms with inflammation. Ann Allergy Asthma Immunol. 2009;103:401‐406. 19927538 10.1016/S1081-1206(10)60359-6

[70] Hofmann AM , Scurlock AM , Jones SM , et al. Safety of a peanut oral immunotherapy protocol in peanut allergic children. J Allergy Clin Immunol. 2009;124:286‐291. 19477496 10.1016/j.jaci.2009.03.045 PMC2731305

[71] Sugnanam KK , Collins JT , Smith PK , et al. Dichotomy of food and inhalant allergen sensitization in eosinophilic esophagitis. Allergy. 2007;62:1257‐1260. 17711545 10.1111/j.1398-9995.2007.01454.x

[72] Duffey H , Peterson K , Firszt R . Population‐based study suggests strong genetic association between eosinophilic esophagitis and asthma. J Allergy Clin Immunol. 2016;137:AB400.

[73] Peterson K , Fang JC , Firszt RA . Familial risk of eosinophilic gastrointestinal disorders (EGID) and atopy in eosinophilic esophagitis (EoE). Gastroenterology. 2015;148:S‐414.

[74] Mansoor E , Cooper G . Epidemiology of eosinophilic esophagitis in the United States: a population based study. Am J Gastroenterol. 2015;110:S693‐S694.26436814

[75] Zafra MP , Cancelliere N , Rodríguez Del Río P , et al. Misregulation of suppressors of cytokine signaling in eosinophilic esophagitis. J Gastroenterol. 2013;48:910‐920. 23229770 10.1007/s00535-012-0723-8

[76] Dellon ES , Gibbs WB , Fritchie KJ , et al. Clinical, endoscopic, and histologic findings distinguish eosinophilic esophagitis from gastroesophageal reflux disease. Clin Gastroenterol Hepatol. 2009;7:1305‐1313. 19733260 10.1016/j.cgh.2009.08.030 PMC2789852

[77] Spergel JM . An allergist’s perspective to the evaluation of eosinophilic esophagitis. Best Pract Res Clin Gastroenterol. 2015;29:771. 26552776 10.1016/j.bpg.2015.06.011 PMC4641822

[78] Langan SM , Irvine AD , Weidinger S . Atopic dermatitis. Lancet. 2020;396:345‐360. 32738956 10.1016/S0140-6736(20)31286-1

[79] Cevhertas L , Ogulur I , Maurer DJ , et al. Advances and recent developments in asthma in 2020. Allergy. 2020;75:3124‐3146. 32997808 10.1111/all.14607

[80] Dellon E , Peterson K , Murray J , et al. Anti‐Siglec‐8 antibody for eosinophilic gastritis and duodenitis. N Engl J Med. 2020;383:1624‐1634. 33085861 10.1056/NEJMoa2012047 PMC7600443

[81] Schanin J , Gebremeskel S , Korver W , et al. A monoclonal antibody to Siglec‐8 suppresses non‐allergic airway inflammation and inhibits IgE‐independent mast cell activation. Mucosal Immunol. 2021;14:366‐376. 32814824 10.1038/s41385-020-00336-9 PMC7946634

[82] Rossi CM , Lenti MV , Di Sabatino A . The need for a reliable non‐invasive diagnostic biomarker for eosinophilic oesophagitis. Lancet Gastroenterol Hepatol. 2022;7:202‐203. 35150646 10.1016/S2468-1253(21)00468-4

